# Training zones in competitive swimming: a biophysical approach

**DOI:** 10.3389/fspor.2024.1363730

**Published:** 2024-03-18

**Authors:** Ricardo J. Fernandes, Diogo D. Carvalho, Pedro Figueiredo

**Affiliations:** ^1^ Centre of Research, Education, Innovation and Intervention in Sport (CIFI2D) and Porto Biomechanics Laboratory (LABIOMEP), Faculty of Sport, University of Porto, Porto, Portugal; ^2^ Physical Education Department, College of Education, United Arab Emirates University, Al Ain, United Arab Emirates

**Keywords:** physiology, biomechanics, intensity, swimmer, training areas

## Abstract

Since swimming performance depends on both physical conditioning and technical proficiency, training zones should be built based on physiology and biomechanics inputs to dispose of structured and effective training programs. This paper presents a zone-based swimming training, supported by the oxygen uptake ($\dot{V}$O_2_) kinetics at low, moderate, heavy, severe and extreme intensities concurrently with lactate and heart rate values. Since technique is vital for efficiently moving through the water, upper limbs frequency and length should also be targeted during the workouts. The index of coordination was also added to our proposal since upper limbs synchronization is a key technical factor. To better establish and characterize a wide range of swimming intensities, the training methods and corresponding contents that better fit each training zone will be suggested. It will be shown that when under/at the anaerobic threshold (at low-to-moderate intensities), swimmers are at homeostasis and can maintain stable internal and external load indicators. However, above that boundary (at heavy and severe intensities), the physiological stable state is no longer observed and the anaerobic metabolism starts contributing significantly, with a technical degradation being more evident when performing near/at the $\dot{V}$O_2max_ intensity. Then, when performing above aerobic power, on typical anaerobic intensities, $\dot{V}$O_2_ kinetics presents a very evident fast rise, ending abruptly due to exhaustion caused by muscle acidosis. This overall knowledge allows advancing toward more objective training programs and highlights the importance of systematic training control and swimmers' evaluation and advice.

## 1 Introduction

Swimming is a sport where participants attempt to cover a specific distance in the shortest possible time and, due to its characteristics, has a strong influence from both physiological and biomechanical variables (1). Since competitive swimming races last from ∼20 s to 15 min (the 50 and 1,500 m events, respectively), it becomes evident that swimmers’ exertions rely on well-developed aerobic and anaerobic energy pathways (2–6). Thus, the importance of monitoring the training process to increase the available quantity and quality of information is unquestionable (7–10). The aerobic and anaerobic energy sources have been traditionally evaluated using $\dot{V}$O_2_ and blood lactate concentrations [La^−^], and heart rate (HR) has been a complementary indicator mainly used in practical settings where expensive instruments and complex procedures are challenging. The assessment of the ATP-CP contribution in swimming has not been a priority because the phosphagen stores have a small capacity, present low relevance in most of the competitive events (11) and muscular biopsy is an invasive procedure very hard to be conducted (12–14).

Swimming technique is also a critical performance determinant (15–18), as displayed in the well-known biophysical expression that exhibit that the maximum velocity attained in a particular context equals the product of the energy input by the ratio between the propulsive efficiency of the biomechanical system and the hydrodynamic drag opposed to the swimmer displacement (19, 20). Swimming can be seen as a thermodynamical process, where energy is processed in each instant of time until a mechanical work is performed with a given energy efficiency (17, 21). However, since the human body has a very low propelling efficiency, slight technical changes positively contributes to performance (8, 11, 22). As swimming technique evaluation is complex, researchers have been assessing stroke frequency (SF) and length (SL), and stroke index (SI), for a long time to observe if a measurable change in $\dot{V}$O_2_, [La^−^] or HR may well reflect technical variability (23, 24). More recently, measuring the lag time between propulsive phases of upper limbs (the Index of Coordination—IdC) became popular for characterizing the coordination patterns during swimming (25). In the current perspective, we present a zone-based swimming training supported by the $\dot{V}$O_2_ behaviour at low, moderate, heavy, severe and extreme intensities, concurrently with [La^−^]and HR values. Also, SF, SL and IdC will be described for each zone due to their biomechanical relevance.

## 2 Establishment of training zones based on relevant swimming determinants

The establishment of accurate training zones for increasing performance in individual, cyclic and continuous sports like swimming has been conducted for many years and it seems evident that the training process should reflect the demands of the races (1, 2, 22). For that purpose, swimmers' monitoring has become more frequent, growing in importance with the appearance of portable $\dot{V}$O_2_ apparatus, [La^−^] analysers, HR monitors and waterproof cameras (10, 11, 26). However, only a single value of one (or more) variable(s) has been used to define a training zone or characterize a swimming event. In addition, studies usually present $\dot{V}$O_2_, [La^−^] or HR maximal values and the mean SF and SL scores in a single bout or incremental protocols, not considering their behaviour along the entire exercise duration. This could be due to the difficulties in assessing internal and external load variables while swimming in standard pools and to the low number of available swimming flumes. Furthermore, even if the general kinematical variables are more easily obtainable by counting the number of cycles per lap or using a chronofrequencemeter, the digitalization of anatomical body parts is more rigorous but very time-demanding, leading to the analysis of only one swimming cycle in a single lap as representative of the entire exercise [e.g., (17)].

**Figure 1:**
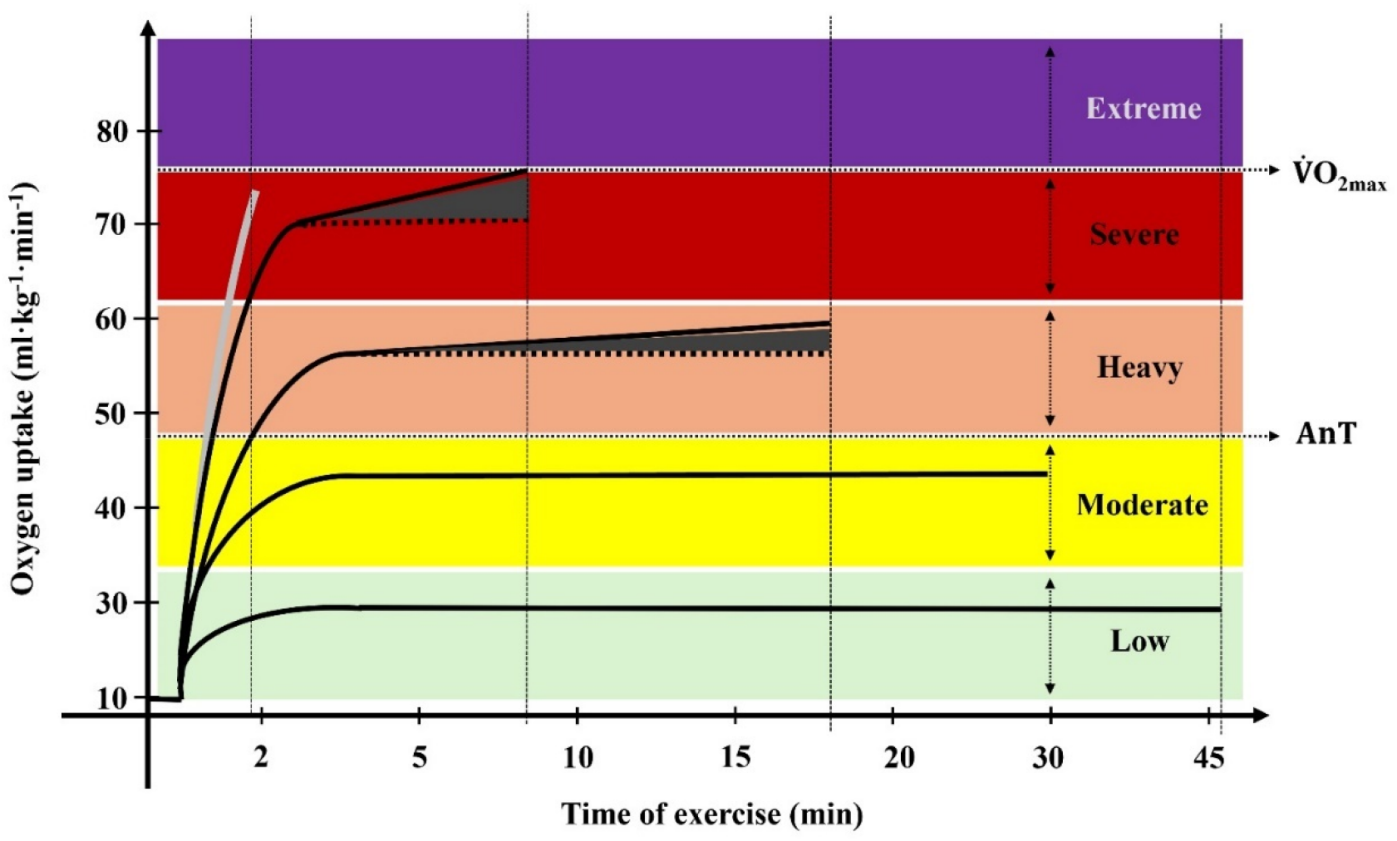
Diagram of the oxygen uptake kinetics at different intensities, with the anaerobic threshold (AnT) and the maximum oxygen uptake ($\dot{V}$O_2max_) being identified. Values were obtained in front crawl swimming.

The detailed behaviour changes of relevant physiological and biomechanical variables with the swimming intensity variation would allow a more accurate definition of the training zones. $\dot{V}$O_2_ assessment in swimming has been implemented regularly only since the 1970s due to previous methodological difficulties (e.g., the incapacity to follow the swimmer along the pool, the difficulty in transporting equipment and the added drag imposed by the respiratory snorkel (27). With the development of $\dot{V}$O_2_ assessment portable and automated systems at the beginning of the current century, it became possible to obtain real-time values during swimming, not only during the recovery phase after exercise (26, 28, 29). Even if the specific $\dot{V}$O_2_ dynamics at the low, moderate, heavy, severe and extreme intensity domains have been mainly centred on running and cycling in laboratory settings (2, 30, 31), some studies conducted a breath-by-breath analysis of well-trained swimmers performing in competition-like conditions. As displayed in Figure 1, at low and moderate swimming intensities, $\dot{V}$O_2_ is characterized by a fast rise (after a non-expressive cardiodynamic phase) that will be prolonged until a steady-state is achieved (9, 32, 33). Since exercise is conducted at paces below or at the anaerobic threshold (AnT), the aerobic energy system supports (almost all) the energy requirements (13), the [La^−^] are low and the effort can be maintained for 30 min or longer (33, 34), with swimmers communicating a very light/light and moderate perception of effort (35–37).

When the swimming intensity exceeds the physiological steady state and the swimmer cannot maintain body homeostasis, $\dot{V}$O_2_ fast component speeds up and the $\dot{V}$O_2_ plateau is no longer observed (32, 33, 38). This is a poorly known training zone that corresponds to the heavy intensity domain, displaying power outputs above the AnT and starting to cause a significant accumulation of [La^−^] over time (34, 39, 40). Here, a notable $\dot{V}$O_2_ slow component leads to an elevated $\dot{V}$O_2_ response (27, 32, 41) and exercise is expressed as hard to accomplish (28, 35). The appearance of this phenomenon, superimposed upon the $\dot{V}$O_2_ fast component, is due to the progressive recruitment and activation of fast twitch glycolic fibres (38, 42, 43), but this needs to be further studied particularly in swimming. The limit separating the heavy from the severe intensity domains is poorly defined in swimming, but the critical power is suggested for other cyclic sports [e.g., (44, 45)]. This has been debated since, by definition, critical power represents the exercise intensity that can be sustained without fatigue, with exhaustion occurring after 30–60 min of exercise (46).

In the severe intensity domain, the exercise intensity is significantly higher than at the AnT, and neither $\dot{V}$O_2_ nor [La^−^] can be stabilized (32, 40, 47), showing a pronounced magnitude compared with heavy intensity efforts (30, 32, 48). During constant pace exercises at this intensity, after an exuberant $\dot{V}$O_2_ fast component caused by the body's need for oxygen as the exercise proceeds, it continues moderately to increase until the point of exhaustion (11, 30, 45). In fact, the $\dot{V}$O_2_ slow component has been commonly reported for heavy (28, 38, 43) but mainly for severe swimming intensities (14, 41, 49) where its magnitude can exceed 1 L.min^−1^ and represent ≥25% of the total $\dot{V}$O_2_ increase above the pre-exercise baseline (50). At this exercise intensity domain, $\dot{V}$O_2max_ is reached, reason why it corresponds to the aerobic power training zone (7), which is perceived as a very hard effort (35, 51). So, similarly to the AnT, the $\dot{V}$O_2max_ is also a critical factor for establishing boundaries between intensity domains.

When swimming above the $\dot{V}$O_2max_ intensity, i.e., at the extreme intensity domain (also known as the anaerobic capacity training zone), despite the $\dot{V}$O_2_-related studies conducted in field conditions are scarce, it was observed that $\dot{V}$O_2_ kinetics only present a fast component, rising in a very fast way and ending abruptly due to exhaustion (29, 41). This domain was the last to be proposed (52) and accounts for short duration and maximal intensity efforts at power outputs where exhaustion occurs before $\dot{V}$O_2max_ is attained (41, 52). Here, there is not enough time to reach $\dot{V}$O_2max_, although the $\dot{V}$O_2_ obtained is of relevant magnitude for such short exercise durations (6, 29). This intensity domain is very scarcely studied in swimming, which does not make sense because the 50 m–200 m events (efforts in-between ∼20 s and 2 min duration) involve the use of different metabolic pathways compared to those involved in longer swimming events (5, 17, 53). [La^−^] are much more expressive than in previous intensity domains (41), with values of ∼16 mmol.L^−1^ at the 100 and 200 m exertions in anaerobically trained swimmers (53, 54). This expresses the use of the glycolysis at its maximal rate, with swimmers struggling until the end of the training set or competitive event with an extremely hard perceived exertion (35, 36).

HR assessment in swimming has been used to control the intensity of the training sets by the neck or wrist palpation during the first seconds of recovery (3). Since the carotid and radial arteries are not easily found, it is hard to count accurately and some rest periods are very short, swimming HR began to be frequently assessed using the easy-to-wear and low-cost telemetric HR monitors. Research-related data shows that HR remains stable at low and moderate swimming intensities within the first few min after the onset of exercise, similarly to $\dot{V}$O_2_ and [La^−^] behaviour (39, 55). In prolonged exercise, HR could progressively rise, particularly if at moderate intensities (34, 39). HR displays a fast rise at the beginning of heavy swimming intensities events, followed by a slight increase or progressive stabilization towards the end of exercise (55, 56). At severe exertions, after a first fast HR rise in the first min of exercise, a progressive levelling off seems to occur but with values significantly higher than in the previous intensity domain (18, 34, 43). At extreme swimming efforts, HR increases rapidly (∼90% of the maximum) during the initial stages of the bouts and climbs progressively toward the maximum as the efforts proceed (56, 57).

A key consideration when using $\dot{V}$O_2_, [La^−^] and HR data is that physiological responses should be related to swimming mechanics (16–18, 58). Since velocity is the product of SF and SL, researchers have always been interested in its assessment to observe if a measurable change in physiological stress-related variables may well reflect technical changes (11, 20). Early investigations on the topic in competitive swimming overestimated SL due to the assumption that it equalled the ratio between velocity and SF, calculating the former based on distance divided by time [e.g., (23, 24)] not accounting for the dive start, variations in mid-pool velocity and turning times. Later, SF and SL were calculated for every 25/50 m race partial in the clean swimming section using a chronofrequencemeter or video/computer analysis (59–61). It is well described that both variables change with intensity, with an increase of SF and a decrease of SL being particularly evident after the AnT intensity (8, 40, 61, 62). SF and SL changes in a non-linearly way (61–63), impacting on how to coordinate contralateral upper limb actions (64, 65). SI was defined as the product of the average swimming velocity and SL, and is considered a valid indicator of swimming efficiency (66). Due to text length constraints this variable will not be further explored but values are available elsewhere [e.g., (3)].

The upper limbs coordination is a fundamental aspect of swimmers' expertise, revealing catch-up, opposition and superposition distinctive coordination modes (<0, = 0 and >0, respectively) in front crawl (25). There is an IdC increase with the intensity rise (18, 65), progressing from catch-up to opposition and superposition coordination modes (18, 64). This evolution mirrors swimmers' adaptive responses to task-specific constraints, reflecting the intensity at which the task is executed (65, 67). There is a relationship between the general kinematical variables and the inter-arm coordination (67, 68), with data suggesting that energy requirements influence the biomechanical characteristics at the mentioned intensities (16, 58, 62, 65). Achieving optimal coordination needs synchronized movements of both upper and lower limbs to enhance velocities and efficiency, with the goal of attaining a 1:3 ratio between the number of upper and lower limb actions, regardless of velocity (69, 70). Consistency in limb coordination is paramount across successive cycles to maintain propulsion and body balance effects, being essential that the lower limb actions occur at the same relative time in each cycle (ensuring the consistent production of desired effects). The widely acknowledged optimal 1:3 frequency ratio emphasizes the importance of synchronized and coordinated actions, and signifies a smooth force-to-time profile, ultimately minimizing intra-cycle velocity variation (70).

Based on the dynamics of the physiological and biomechanical variables described in the above-referred literature, the following paragraphs, and our own knowledge and experience, different training zones are displayed in Table 1. It is possible to observe that the AnT (assessed metabolically using [La^−^] and the $\dot{V}$O_2max_ (evaluated through ventilatory data) are critical factors for establishing boundaries between intensity domains. In this field, SF might serve to delineate the boundary between moderate and heavy intensity domains when [La^−^] is inaccessible (8, 40, 65) and HR combined with the rating of perceived exertion (RPE) can establish a boundary between heavy and severe intensity domains (35, 51, 62). When not disposing of ventilatory data, the 8 mmol.L^−1^ of [La^−^] might be used for limiting the upper boundary of the severe intensity domain since it is an average value commonly used for characterizing aerobic power swimming efforts (34, 57).

**Table 1:** Competitive swimming training zones (and corresponding methods and contents) based on commonly assessed physiologic and biomechanical variables (to be used, preferably, in populations of front crawl high-level swimmers).

Training zones	Intensity domains	Boundaries	$\dot{V}$O_2_ (mlkg^−1^·min^−1^)	[La^−^] (mmol.L^−1^)	HR (beats·min^−1^)	SF (cycles·min^−1^)	SL (m·cycle^−1^)	IdC (−1 – 1)	RPE (6 – 20)	Time (min)	Training methods	Training contents
Aerobic capacity 1	Low intensity	Upper: AnT	Monoexponential rise and steady state in ∼3 min	No increase (<2/3)	<140 – 50	<30	2.5 – 3.0	< 0	<10	≥40	Long duration method/Extensive method	Continuous exercise/Long duration exercise with intervals
Aerobic capacity 2	Moderate intensity	Around the AnT	Monoexponential rise and steady state in ∼3 min	Transient increase (<3/4)	<150 – 60	30 – 35	2.2 – 2.9	<0	11 – 12	∼30	Long duration method/Extensive and interval methods	Continuous exercise and fartlek/Long duration exercise with intervals
Aerobic capacity 3	Heavy intensity	Lower: AnT	Biexponential rise, with a slow component superimposed on primary phase	High (4/5 – 7)	160 – 70	35 – 40	2.1 – 2.7	<0	13 – 15	10 – 20	Intensive interval method/Competitive method	Medium and short duration exercise with intervals/Fractioned race pace
Aerobic power	Severe intensity	Upper: VO_2max_	Biexponential rise, with a slow component developing until VO_2max_	Very high (8 – 10)	170 – 90	40 – 45	2.0 – 2.5	≤0	16 – 17	3 – 10	Intensive interval method/Competitive method	Medium and short duration exercise with intervals/Fractioned race pace
Anaerobic power and capacity	Extreme intensity	Lower: VO_2max_	Fast component rise without achieving VO_2max_	Utmost > 12	>190	>45	1.9 – 2.5	≥0	18 – 20	<2/3	Intensive interval method/Repetitions method/Competitive method	Medium and short duration exercise with breaks/Complete recovery intervals/Fractioned race pace

When the aim is to target a specific intensity domain or training zone, certain considerations regarding training methods and contents should be recognized to better structure the swimming sets. For low and moderate intensities, the most effective approach is to employ continuous exercise with extended durations [or extensive interval training with short rests (66, 71, 72)], emphasizing the importance of maintaining high technical quality when maintaining a steady pace (14, 15, 22) or even with slight changes of intensity [e.g., the fartleck (3, 13)]. The development of aerobic capacity 1 and 2 are more relevant in early stages of the season and in younger/less experienced swimmers (58), aiming to increase the systemic oxidation capacity of pyruvic acid, lactic acid and lipids (7, 11). The aerobic capacity 1 training zone obtain high percentages of the total training volume since it is used for warming up and cooling down in each training session, as well as for promoting recovery between intense bouts (13, 22, 71). Aerobic capacity 3 implies increasing the intensity of the training sets, using the intensive interval training method (3) and, in the competitive period, breaking the so-called long-distance events in laps by employing the fractioned race pace training strategy (e.g., 15 × 100 m at 1,500 m pace with 10 s intervals).

The aerobic power training zone focuses on the traditionally called medium-distance races like the 400 m (26, 49) and aims to increase the transport, diffusion and peripheric perfusion of O_2_, as well as the mitochondrial capacity (73). The depletion of the muscle glycogen stores will not allow the training series to go over 10 min of duration if conducted at $\dot{V}$O_2max_ pace (as it should). In the competitive period, the 400 m pace can be trained by making 4 × 100 m (10 s rest) at race pace (or even faster). Last but not least, the anaerobic power and capacity zones, as a fundamental focus of attention of competitive swimming (10, 11, 15), aims to activate the activity of glycolytic enzymes and reduce their sensitivity to metabolic acidosis by increasing buffering capacity (7, 71, 74). Since long and slow distance training can compromise sprint performance (11, 22), the swimmers’ engagement at extreme exertions needs to be maximal. Moreover, a wise definition of the rest period duration is the key to success since central and peripheral fatigue is a limiting factor of the training series continuity (3, 15). Early in the program, workouts should propose higher intensities for swimmers already fatigued and, later on, move on to conduct training sets in a fresh state to facilitate higher speeds (8, 58).

## 3 Discussion

In the large spectrum of swimming intensities, the most well-known and manageable are those supported by the aerobic energy system, i.e., the low and moderate efforts (typically the 3–10 km open water events). However, above the AnT, exercise cannot be maintained for long due to the significative contribution of the anaerobic metabolism, with fast twitch fibres being progressively recruited (3, 13). Thus, fatigue will appear sooner or later when swimming at heavy intensities, with this being considered a “grey training zone” (where the 800 and 1,500 m races are included). The severe intensity domain is reached at exertions close to $\dot{V}$O_2max_ paces, with the 400 m events being accepted as typical efforts. Exertions are very strenuous at the 50, 100, and 200 m competitive swimming events that are situated in the extreme intensity domain. Thus, anaerobic capacities are decisive to perform well and should be fully developed in addition to aerobic qualities (8, 10, 15). The evidence reported suggests that the behavior of relevant physiological variables should be well-considered when establishing training zones but always concurrently with upper limbs' general kinematics and coordination (1, 20). In fact, to succeed well, swimmers should not only focus on physical conditioning but also on developing technical skills (11, 22, 75). By combining training intensities, it is possible to optimize performance better, reduce injury risk and prevent overtraining, justifying the proposal of a new paradigm concerning the traditionally used training zones.

## Data Availability

The original contributions presented in the study are included in the article/Supplementary Material, further inquiries can be directed to the corresponding author.
